# A phosphoprotein gene RT-qPCR assay for detecting all lineages of peste des petits ruminants virus

**DOI:** 10.3389/fmicb.2025.1638778

**Published:** 2025-07-24

**Authors:** Jiao Xu, Yonggang Zhao, Huicong Li, Qinghua Wang, Jiarong Yu, Yingli Wang, Jingyue Bao, Zhiliang Wang

**Affiliations:** ^1^Exotic Herbivore Disease Surveillance and Research Center, China Animal Health and Epidemiology Center, Qingdao, China; ^2^College of Veterinary Science, Qingdao Agricultural University, Qingdao, China

**Keywords:** PPRV, RT-qPCR, diagnosis, P gene, lineage

## Abstract

Peste des petits ruminants (PPR) is a highly contagious disease caused by peste des petits ruminants virus (PPRV). PPRV is classified into four lineages based on the nucleocapsid (N) or fusion (F) genes. We established a TaqMan quantitative real-time reverse transcription polymerase chain reaction (RT-qPCR) assay using a pair of primers and a probe based on the phosphoprotein (P) gene. The assay was assessed for its sensitivity, specificity, and repeatability in the detection of field samples and was compared with the standard detection method. The developed method could detect all lineages of PPRV, with a sensitivity of four copies/μL for lineage II to IV and 40 copies/μL for lineage I. The results of the specificity test indicated that only the different lineages of PPRV could be detected using the developed method, with no cross-reaction observed with other viruses. The coefficients of variation for both intra-assay and inter-assay repeatability tests were all below 1.50%, demonstrating good repeatability. The detection of field samples, including PPRV-positive and PPRV-negative samples, indicated that all samples were detected correctly, showing a high concordance with the standard detection method. The developed method could detect PPRV with a lower cycle threshold value compared to the previously established N gene-based method, especially for weakly positive samples. This RT-qPCR assay provides a valuable tool to facilitate targeted surveillance and rapid differential diagnosis in regions with an active circulation of PPRV, enabling timely epidemiological investigations and strain-specific identification.

## Introduction

Peste des petits ruminants (PPR) is a highly contagious disease caused by peste des petits ruminants virus (PPRV). It mainly affects small ruminants, including sheep, goats, and wild animals. Several symptoms, such as fever, increased discharges, gastritis, diarrhea, and pneumonia, are usually observed in infected animals (Rahman et al., 2020). PPRV can be classified into four lineages according to the N or F gene. Lineages I and II were mainly discovered in Africa; lineage III was mostly distributed in Africa and the Arabian Peninsula; and lineage IV was the most widely distributed among all lineages, occurring across Africa, Asia, and Europe (Niu et al., 2022; Parida et al., 2024). Huge economic losses to the animal breeding industry are caused by PPR each year. Drawing on the success of rinderpest eradication, the World Organisation for Animal Health (WOAH) has identified PPR as a target for global control and eradication by 2030.

PPR was first discovered in Côte d’Ivoire in the early 1940s, and since then, it has steadily spread to more than 70 countries across Africa, Asia, and some parts of Europe. In 2007, China reported its first case of PPR in Tibet. In 2013, PPR re-emerged in the Xinjiang Uygur Autonomous Region and rapidly spread to almost all provinces. The number of PPR cases reached a peak in 2014. Since then, various control methods have been implemented, including culling, restriction of transportation of susceptible animals, compulsory vaccination, and active surveillance (Liu et al., 2018). Benefiting from the measures mentioned above, the PPR pandemic was well controlled. The number of reported cases decreased sharply since the second half of 2014, and only a few PPR cases have been reported in recent years.

The non-specific clinical symptoms of PPR highlight the need for laboratory diagnosis to confirm PPR infection. Serum-based tests [enzyme-linked immunosorbent assays) for the detection of viral antibodies, viral nucleic acid detection (reverse transcription polymerase chain reaction (RT-PCR) and quantitative real-time reverse transcription polymerase chain reaction (RT-qPCR)], and virus isolation are recommended for PPR diagnosis by the WOAH. However, for PPRV isolation, multiple and sequential blind cell passages are required, and it may take up to 2 weeks for a cytopathic effect to appear (Kinimi et al., 2020). In addition, professional skills are required, which may not be available in some developing countries or regions. Furthermore, the time-consuming nature of the test also limits its large-scale application. The application of compulsory vaccination against PPRV in China and some other countries means that PPR cannot be confirmed using serum-based tests, which are more frequently used to evaluate immune effectiveness. However, serum-based methods can still be used for PPR diagnosis in a PPR-free region. Reverse transcription polymerase chain reaction (RT-PCR) and reverse transcription quantitative polymerase chain reaction (RT-qPCR) have become the most widely used methods to detect PPRV, and research has confirmed that compared to RT-PCR, RT-qPCR possesses higher sensitivity and specificity (Qi et al., 2023; Kwiatek et al., 2010). Previously, we developed an RT-qPCR method based on the N gene, which could detect 8.1 copies of PPRV RNA per reaction mixture (Bao et al., 2008). However, *in silico* assessment of the PPRV RT-qPCR assay indicated that its sensitivity was lower than other RT-qPCR methods, especially for lineage II (Flannery et al., 2019; Kwiatek et al., 2010). A previous study found that the vaccine strain of PPRV (Nigeria 75/1) could be detected in goats after vaccination, although no viral transmission was confirmed (Milovanović et al., 2023). Based on this observation, a differential RT-qPCR method for PPRV was also established (Li et al., 2016). In addition, various novel diagnostic methods have been established in recent years, including reverse transcription loop-mediated isothermal amplification (Li et al., 2010), reverse transcription isothermal recombinase polymerase amplification (Li et al., 2018), and lateral flow strip reverse transcription isothermal recombinase polymerase amplification assays (Yang et al., 2017), all reported to possess higher sensitivity than conventional RT-PCR. The results were also conveniently observed and determined; however, their high cost has limited widespread application.

The majority of RT-qPCR methods for PPRV have been established based on the N gene because of its proximity to the viral promoter region, which is responsible for the N protein’s status as the most abundantly expressed viral protein during replication (Kumar et al., 2014; Batten et al., 2011). In contrast, the P gene is rarely selected as the target. However, based on our sequence alignment results, several conserved regions were identified in the P gene, indicating its potential as a novel target for PPRV detection. This finding not only expands the target selection for PPRV diagnosis but also provides new insights for developing alternative detection methodologies. In addition, most existing RT-qPCR assays for PPRV detection were developed several years or even over a decade ago, and their detection efficiency may have declined for some newly emerged variant strains. To address this limitation, we aligned all currently available PPRV sequences worldwide and specifically designed this assay to maximize its detection efficiency for currently circulating strains across different regions. This approach effectively compensates for the shortcomings of previously established detection methods. In this study, we established a P gene-based RT-qPCR method, systematically analyzed its sensitivity, specificity, and repeatability, and compared it with our previously established N gene-based method.

## Materials and methods

### Primer and probe design

Full genomic sequences of PPRV were collected from GenBank and aligned using SnapGene software (GSL Biotech LLC, Boston, MA, United States). The primers and probe were designed based on the conserved regions of the PPRV P gene. The forward primer is 5′-GAYTCCGAGTATGAGTATGAGGATG-3′, the reverse primer is 5′-GCTATCATTATACTGGAMAGATGGCC-3′, and the TaqMan probe is FAM-CGTGCAAGCATTGCYAARATCCATGA-BHQ1.

### Virus and plasmids

Goat pox virus, orf Virus, foot-and-mouth disease virus, PPRV China/XJYL/2013, and PPRV Nigeria 75/1 were acquired from the National Reference Laboratory for Peste des Petits Ruminants (the China Animal Health and Epidemiology Center, Qingdao, China). Partial P genes from PPRV/Cote_dIvoire/1989 (lineage I), PPRTV/Nigeria 75/1 (lineage II), PPRV/KN5/2011 (lineage III), and PPRV China/XJYL/2013 (lineage IV) were commercially synthesized and cloned into vector pUC57. All plasmids were constructed and verified by Shanghai Sangon Biotechnology Company (Shanghai, China) for use in sensitivity and repeatability tests.

### RNA extraction and real-time quantitative RT-PCR

All samples were processed in a biosafety level III (BSL-3) laboratory. Viral RNA was extracted using a magnetic bead-based viral DNA/RNA extraction kit (Xi’an Tianlong Science and Technology Co., Ltd., Xi’an, China), following the manufacturer’s instructions. RT-qPCR amplification and detection were performed using a Bio-Rad PCR machine (Hercules, CA, United States) and a one-step RT-qPCR kit (RR600A, Takara, Shiga, Japan). Each 25 μL reaction mixture contained 12.5 μL of one-step PrimeScript III RT-qPCR Mix (2×), 1 μL of the forward primer (10 μM), 1 μL of the reverse primer (10 μM), 0.5 μL of the probe (10 μM), 3 μL of the RNA sample, and 7 μL of RNA-free water. All samples were tested in duplicate. The cycling conditions were set as follows: reverse transcription at 50°C for 10 min, reverse transcriptase inactivation and DNA polymerase activation at 95°C for 5 min, and 40 cycles of amplification (denaturation at 95°C for 15 s and annealing at 60°C for 30 s). Fluorescence signals were collected at 60°C in each cycle. Cycle threshold (Ct) values were assigned to each sample in the exponential phase of the amplification plot during each cycle.

### Sensitivity test and standard curve

PPRV/Cote_dIvoire/1989, PPRTV/Nigeria 75/1, PPRV/KN5/2011, and PPRV China/XJYL/2013 plasmids were serially diluted 10-fold to concentrations ranging from 4 × 10^0^ to 10^5^ copies/μL, respectively. The diluted plasmids were used as templates for detection using the designed primers and probe. Standard curves with the logarithm of the viral copy number and the measured Ct value for all lineages of PPRV were constructed.

### Specificity test

The specificity of the method was assessed by detecting various viruses, including goat pox virus, orf virus, and foot-and-mouth disease virus, as well as the PPRV China/XJYL/2013 strain, the PPRV Nigeria 75/1 strain, PPRV/Cote_dIvoire/1989, and PPRV/KN5/2011 plasmids.

### Repeatability test

Plasmids including lineages I to IV of PPRV were serially diluted 10-fold to concentrations ranging from 4 × 10^0^ to 10^5^ copies/μL. Different concentrations of the plasmids were detected using the primers and probe. Inter-assay and intra-assay repeatability were then evaluated.

### Field samples

A total of 50 field samples comprising swabs and tissues were collected in our laboratory. PPRV was inactivated using 1% (w/v) NaOH in a BSL-3 laboratory. Viral RNA was extracted, followed by reverse transcription in a BSL-2 laboratory. All field samples were tested and confirmed as PPRV-positive or PPRV-negative using conventional RT-PCR (Couacy-Hymann et al., 2002) before the primers and probe designed in this study were used to detect the virus in these field samples. All positive samples belonged to PPRV lineage IV.

### Comparison with the previous method

A total of 40 lineage IV PPRV-positive samples, collected during the PPR outbreak in China from 2007 to 2025, with different Ct values were detected using the method developed in this research and an N gene-based RT-qPCR method that we established previously (Bao et al., 2008). The Ct values for each sample were then compared between the two methods.

## Results

### Multiple sequence alignments of the primers and probe

**Figure 1:**
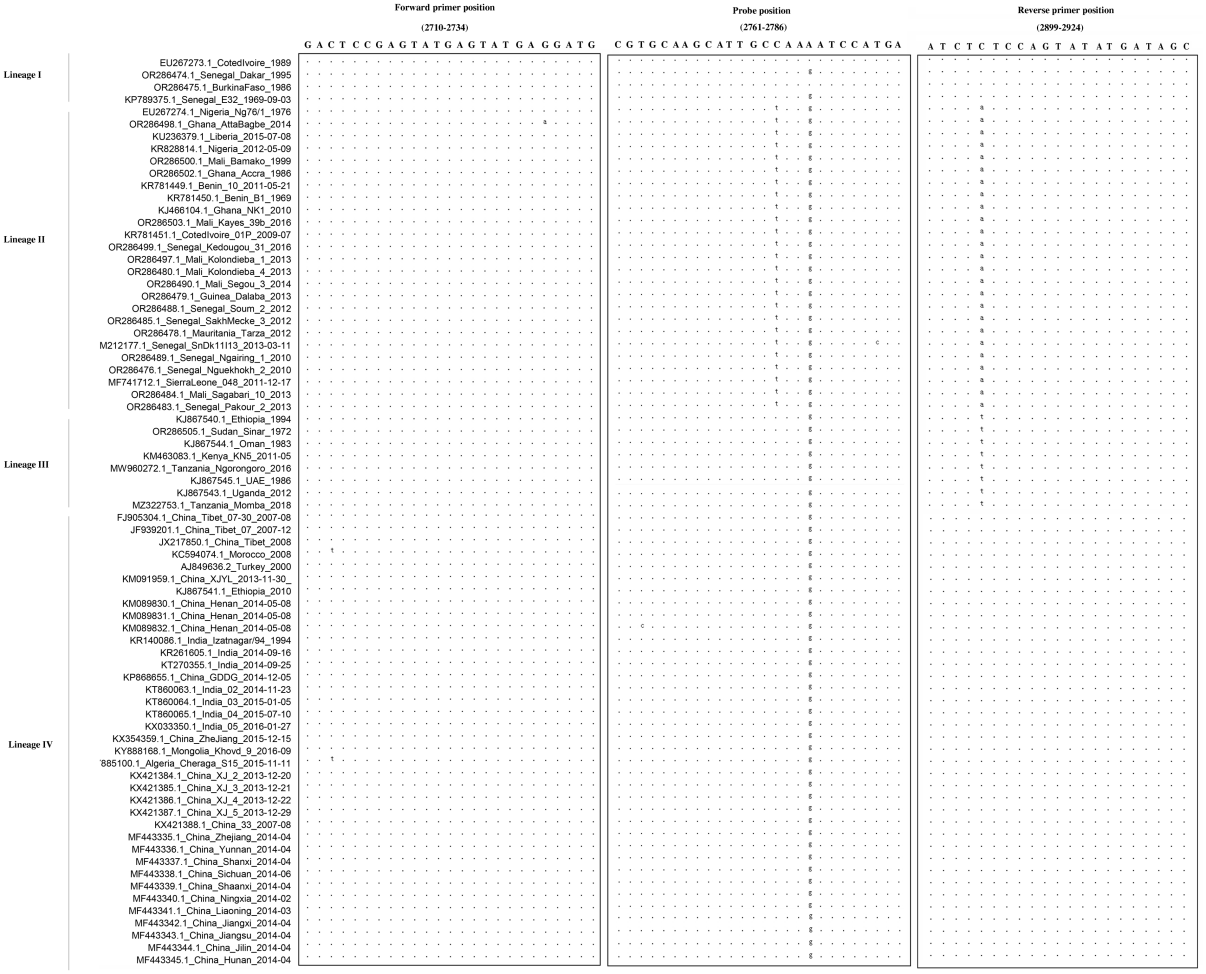
Alignment of the primers and probe targeting the gene across multiple PPRV strains. The sequences of the forward primer, probe, and reverse primer are shown in boxes. Dots represent nucleotides identical to the primers and probe. The designed primers and probe mapped well with most epidemic PPRV strains, indicating that both the primers and probe developed in this study exhibited specificity for conserved genomic regions within all lineages of PPRV.

The results of the primer and probe alignment are shown in Figure 1. The designed primers and probe mapped well with most epidemic PPRV strains, indicating that both the primers and probe developed in this study exhibited specificity for conserved genomic regions within all lineages of PPRV.

### Sensitivity of the developed method

**Figure 2:**
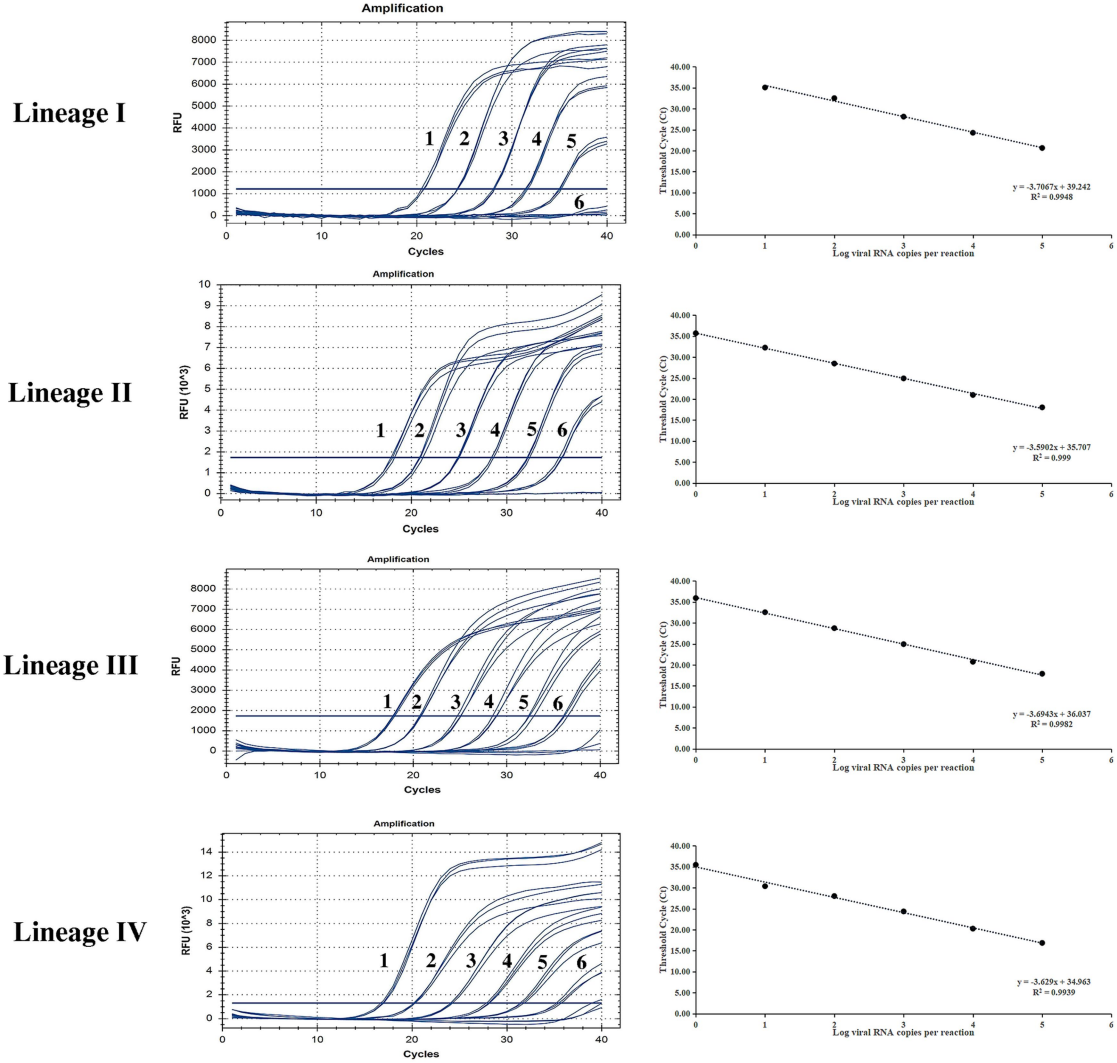
Sensitivity testing and standard curve of the RT-qPCR assay based on the P gene. The lowest copy number for lineage I that could be determined was 40 copies/μL and for lineages II to IV was four copies/μL, demonstrating high sensitivity for all PPRV lineages. The correlation coefficients of the standard curves were all higher than 0.99, demonstrating excellent linearity between concentration and the detection signal (lineage I, 0.9948; lineage II, 0.999; lineage III, 0.9982; lineage IV, 0.9939).

Ten-fold gradient dilutions of the different lineages of PPRV plasmids were detected. According to Figure 2, for lineage I, the minimum template concentration detected by this method was 40 copies/μL, while for lineage II to IV, the minimum template concentration detected by this method was four copies/μL. The dynamic range of the assay over a 10-log-unit span of viral RNA concentrations ranged from four to 4 × 10^5^ RNA copies/μL. A standard curve for the P-gene RT-qPCR assay was also constructed. The correlation coefficients for linages I to IV were all above 0.99, demonstrating excellent linearity between concentration and the detection signal. (lineage I, 0.9948; lineage II, 0.999; lineage III, 0.9982; and lineage IV, 0.9939).

### Results of the specificity test

#### Specificity of the developed method

**Figure 3:**
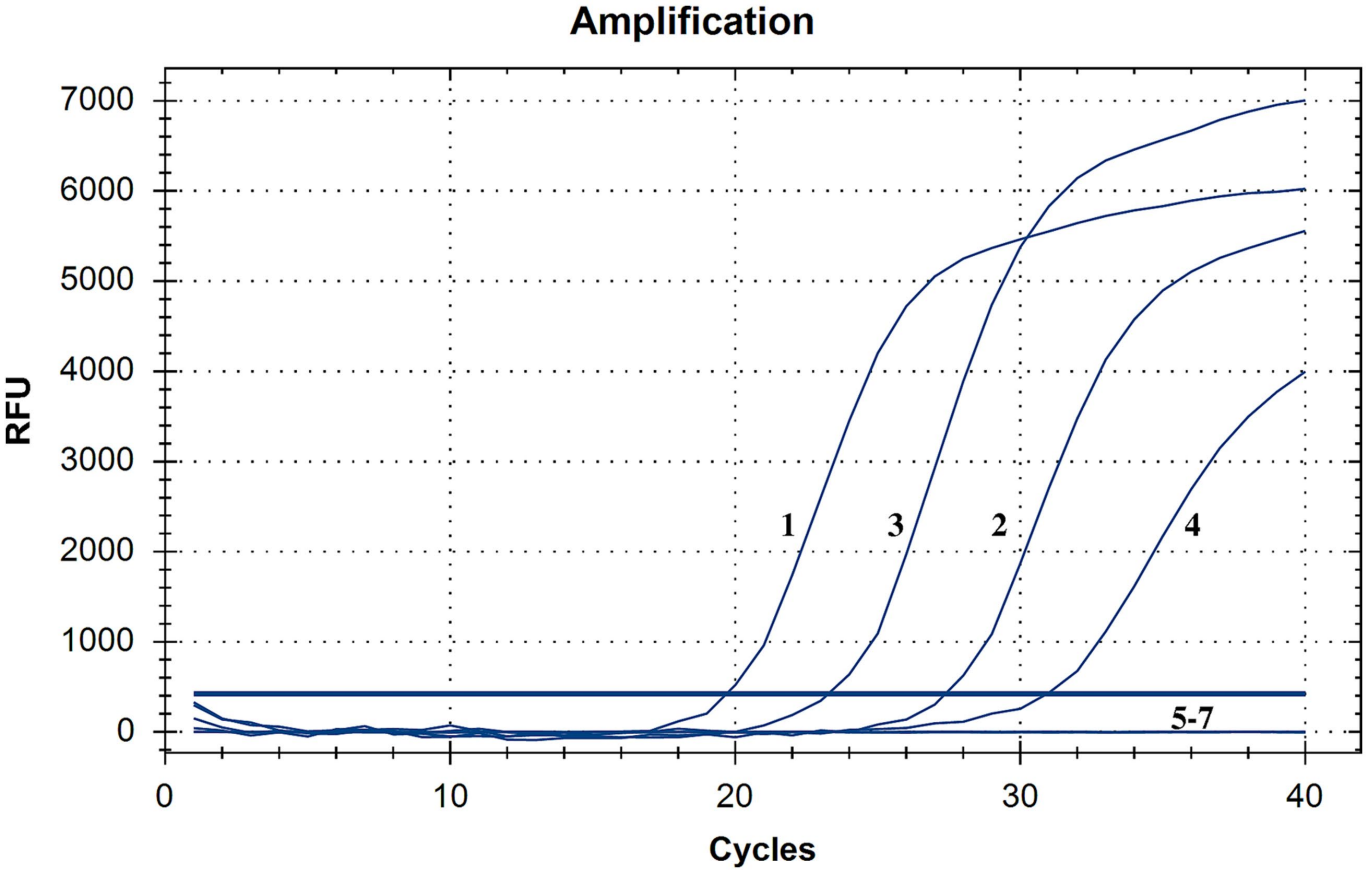
Specificity testing of the RT-qPCR assay. Only PPRV viruses and plasmids were detected with positive fluorescence signals, and no positive signals were observed for other viruses, demonstrating high specificity (1, China/XJYL/2013, 2, KN5/2011, 3, Nigeria 75/1, 4, PPRV/Cote_dIvoire/1989, 5, GPV, 6, ORFV; 7, FMDV).

The results of specificity testing are shown in Figure 3 and Table 1. The results indicated that only different lineages of PPRV viruses and plasmids could be detected by the developed method, and no cross-reaction with other viruses (GPV, ORFV, and FMDV) was observed, ensuring a reliable detection of PPRV.

**Table 1:** Specificity of the developed method.

Number	Name of strains	GenBank accession number	Lineage	Ct value
1	China/XJYL/2013	KM091959	IV	19.70
2	KN5/2011	KM463083	III	27.37
3	Nigeria 75/1	HQ197753	II	23.26
4	PPRV/Cote_dIvoire/1989	EU267273	I	30.94
5	GPV	/	/	—
6	ORFV	/	/	—
7	FMDV	/	/	—

#### Repeatability of the developed method

Different lineages of PPRV were detected for the repeatability test at various concentrations. According to Table 2, for lineages I to III, the coefficients of variation for both inter-assay and intra-assay tests were all below 1.5% (0.11 to 1.13%). For lineage IV, these coefficients of variation were all below 1.0% (0.07 to 0.95%). The results demonstrated the robustness and consistency of the method, which is important for clinical and surveillance applications.

**Table 2:** Repeatability of the developed method.

Lineage I							Lineage II					
Standard copies /μL	Inter-assay repeatability of Ct value			Intra-assay repeatability of Ct value			Inter-assay repeatability of Ct value			Intra-assay repeatability of Ct value		
	Mean	SD	CV (%)	Mean	SD	CV (%)	Mean	SD	CV (%)	Mean	SD	CV (%)
4 × 10^5^	20.88	0.20	0.97	20.42	0.14	0.67	18.24	0.12	0.65	18.11	0.07	0.37
4 × 10^4^	24.17	0.17	0.72	24.18	0.14	0.58	21.15	0.25	1.19	20.98	0.11	0.52
4 × 10^3^	27.90	0.30	1.06	28.63	0.08	0.28	24.53	0.36	1.47	24.87	0.15	0.59
4 × 10^2^	32.87	0.27	0.83	32.74	0.22	0.68	28.79	0.25	0.87	28.75	0.30	1.04
4 × 10^1^	35.25	0.28	0.28	35.53	0.13	0.35	32.41	0.31	0.96	32.41	0.09	0.29
4 × 10^0^	/	/	/	/	/	/	35.53	0.36	1.02	35.83	0.05	0.14

Lineage III							Lineage IV					
Standard Copies /μL	Inter-assay repeatability of Ct value			Intra-assay repeatability of Ct value			Inter-assay repeatability of Ct value			Intra-assay repeatability of Ct value		
	Mean	SD	CV (%)	Mean	SD	CV (%)	Mean	SD	CV (%)	Mean	SD	CV (%)
4 × 10^5^	17.83	0.13	0.75%	17.84	0.06	0.34%	16.68	0.18	1.06%	16.82	0.05	0.31%
4 × 10^4^	20.60	0.20	0.97%	20.40	0.04	0.20%	21.08	0.07	0.31%	20.14	0.04	0.21%
4 × 10^3^	24.56	0.28	1.13%	24.84	0.04	0.16%	24.46	0.17	0.71%	24.61	0.23	0.95%
4 × 10^2^	28.82	0.16	0.57%	28.76	0.21	0.73%	28.11	0.10	0.36%	28.06	0.03	0.11%
4 × 10^1^	32.75	0.24	0.72%	32.81	0.23	0.70%	30.66	0.29	0.95%	30.35	0.23	0.77%
4 × 10^0^	35.96	0.08	0.21%	35.94	0.04	0.11%	35.18	0.23	0.66%	35.34	0.07	0.18%

SD, standard deviation; CV, coefficient of variation.

#### Detection of field samples

**Figure 4:**
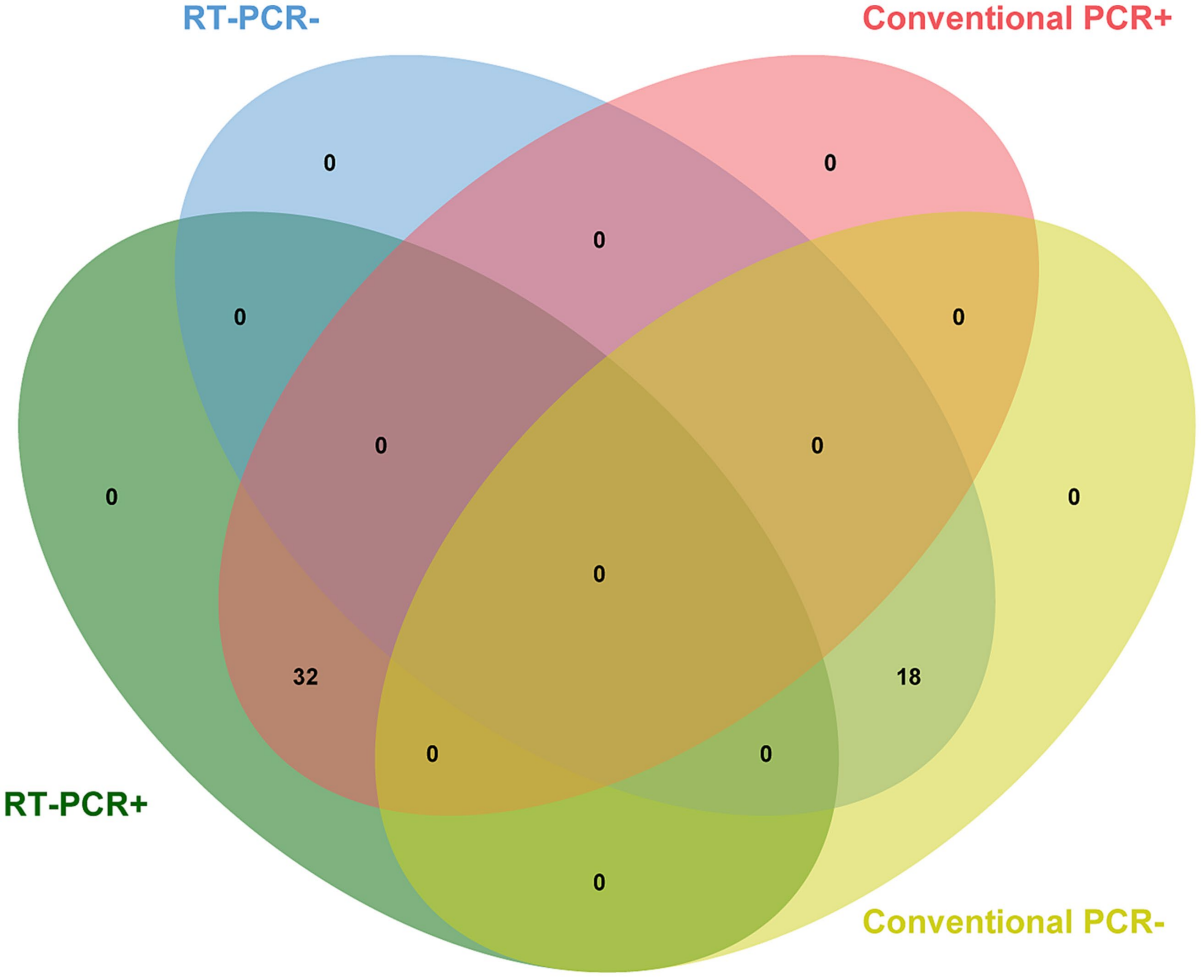
Detection of field samples. All 32 positive samples previously confirmed by conventional RT-PCR were detected successfully using the novel method, demonstrating 100% concordance.

A total of 50 field samples collected from different parts of China were tested using conventional RT-PCR and the developed RT-qPCR methods. According to Figure 4, all 32 positive samples confirmed by conventional RT-PCR were detected successfully using the novel method, and all 18 negative samples were undetectable by both methods, demonstrating 100% concordance.

#### Comparison with a previously developed method

**Figure 5:**
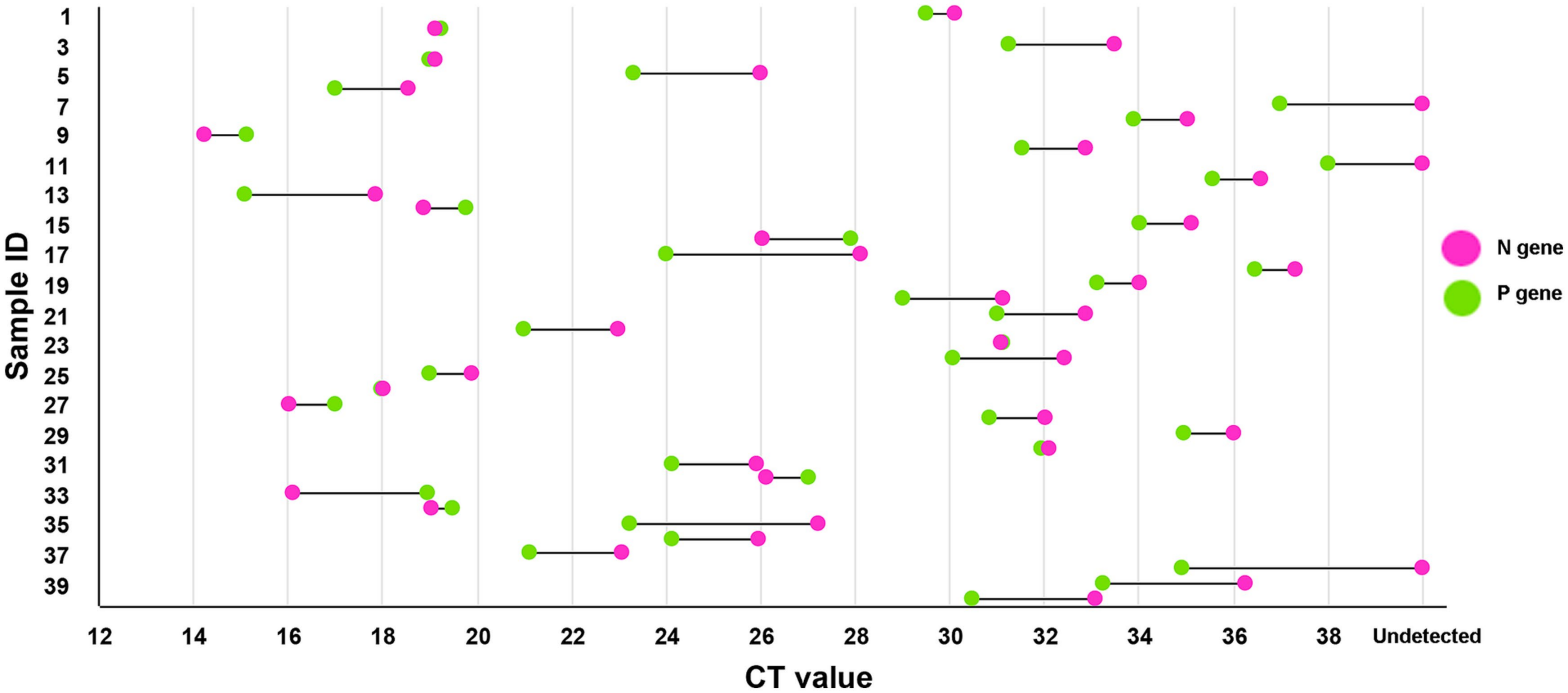
Comparison with the previous method. The method developed in this study could detect PPRV with a lower Ct value in most samples compared to the previously established N gene-based method. Almost all (about 94%) weakly positive samples had improved Ct values, and three samples that could not be detected by the N gene-based method were successfully detected by the new method, demonstrating its higher sensitivity compared to the previous method.

A total of 40 PPRV-positive samples were detected using two RT-qPCR methods—the newly developed method and an N gene-based method we established earlier. All samples were previously confirmed as PPRV positive using the conventional RT-PCR method mentioned above (Couacy-Hymann et al., 2002). According to Figure 5, for the majority of samples (31/40), the new method established in this study detected the virus with lower Ct values compared to the N gene-based method, especially for weakly positive samples. Approximately 94% (18/19) of the weakly positive samples had improved Ct values. Notably, three samples that could not be detected by the N gene-based method were successfully identified using the new method.

## Discussion

Since its initial discovery in Africa, PPRV has spread widely across West and Central Africa, Arabia, the Middle East, southern Asia, and several parts of Europe. The majority of the countries in these regions are developing nations that rely heavily on subsistence farming for food and trade, with small ruminants serving as a vital source of both food and income. The high mortality rate caused by the virus among small ruminants has imposed a heavy burden on related industries. The virus is serologically monotypic; however, it has been classified into four lineages. Lineage I is no longer widely circulating, although research has confirmed the persistence of lineage I PPRV in two regions of Mali at least until 2014 (Tounkara et al., 2021). In addition, no lineage I PPRV strain has been detected worldwide in the past decade. Lineage III PPRV was frequently discovered in Africa. The circulation range of lineage II is wider than that of lineages I and III, which are usually detected in West Africa. Nevertheless, lineage IV has become the main epidemic strain around the world in recent years. Almost all recently confirmed PPRV cases in Asia and Europe have been classified as lineage IV. In China, although the origins of the first PPRV outbreak in Tibet and the second outbreak in Xinjiang were different, all strains derived from China have been classified into lineage IV (Wu et al., 2016). Lineage IV is also replacing other lineages in some African countries (Biguezoton et al., 2024; Couacy-Hymann et al., 2023). All these reports indicate that lineage IV should receive more attention.

The basis of PPR epidemic prevention and control depends on biosecurity measures and monitoring efforts; therefore, a rapid, sensitive, and stable detection method is essential. Several RT-qPCR assays for PPRV detection have been developed, with the limit of detection (LOD) ranging from 10 to 32 genomic copies per reaction (Flannery et al., 2019; Kwiatek et al., 2010; Batten et al., 2011; Polci et al., 2015). As a transboundary animal disease, PPRV can spread to previously unaffected regions through the movement and commercial trade of live animals. Therefore, developing a rapid diagnostic method specific to PPRV is critical for its effective control. Early detection of the virus is crucial to block its transmission. According to our results, the developed method could detect a minimum of four copies/μL of PPRV. Although its sensitivity for lineage I is relatively low, as previously mentioned, no lineage I PPRV strains have been confirmed in the past decade, indicating that their global circulation has effectively ceased. Consequently, while our detection method shows relatively lower sensitivity for lineage I, this limitation has a negligible impact on global PPRV surveillance. Furthermore, for prevalent PPRV strains, the developed method was more sensitive than current RT-qPCR methods; however, further optimization of primers and probes is still required to enhance the detection sensitivity for all PPRV lineages. Indeed, its sensitivity was significantly higher compared to the N gene-based method. In the comparison between the developed method and the N gene-based method, our results indicated that most Ct values generated by the new method were markedly lower, especially for weakly positive samples. This suggests that our method could detect PPRV even at low viral copy levels. Detection of weakly positive samples is particularly significant as these samples may represent animals in the early stages of infection. By improving the detection rate of weakly positive cases, the virus can be identified at the earliest possible stage of transmission. This enables targeted implementation of necessary containment measures to effectively interrupt viral spread. The N gene-based method was developed by us in 2007, when PPR was first introduced in China. In 2013, the PPR virus entered China again, and genetic evolutionary analysis revealed that the viruses from these two introductions belonged to different subclades, although both were classified as lineage IV (Bao et al., 2017). The currently circulating strains in China belong to the same subclade as the virus introduced in 2013 (Xu et al., 2024). Sequence comparison showed that, compared to the 2007 strain, several nucleotide mutations occurred in the probe-binding region targeted by our previously designed assay. This may have led to reduced detection sensitivity for the currently prevalent strains. Compared to the aforementioned new diagnostic methods, our approach still holds an advantage in terms of sensitivity. For instance, the reverse transcription isothermal recombinase polymerase amplification (RT-RPA) assay developed by Li et al. (2018) achieved a detection limit of 14.98 copies/μL for PPRV, while the lateral flow strip-based RT-RPA method established by Yang et al. (2017) demonstrated a similar detection limit of 14.98 copies/μL. Both of these values are higher than our assay’s detection limit. Compared to RT-qPCR, these novel methods are simpler to operate, require shorter reaction times, and do not need specialized instruments. The results are more visually intuitive, making them better suitable for rapid field testing and more user-friendly; however, their higher usage cost (approximately five times that of RT-qPCR per reaction) limits their application in large-scale monitoring. The low coefficient of variation in both intra-assay and inter-assay repeatability tests indicates that the method is stable, which is essential for a robust and practical detection method. The lack of cross-reactivity with other viruses indicates high specificity. To verify the method’s capacity for clinical detection, field samples were detected. The results indicated that the method could detect PPRV in various kinds of samples with high concordance compared to conventional RT-PCR. Notably, only lineage IV strains of PPRV are currently circulating in China (Xu et al., 2024), and obtaining viruses or nucleic acids from other lineages abroad presents significant challenges. As a result, all PPRV-positive clinical samples available were exclusively lineage IV. For the detection of other lineages (I-III), we performed validation using artificially synthesized plasmids. The results confirmed that our method successfully detected all lineage I-III plasmid targets (data not shown).

Due to its operational simplicity and high specificity, RT-qPCR is one of the diagnostic methods recommended by the WOAH for PPRV surveillance. However, as an RNA virus (albeit one with relatively slow mutation rates), nucleotide mutations in primer-or probe-binding regions can still reduce detection efficiency. Therefore, comprehensive comparative analysis of global viral sequences is essential to design updated primers/probes that can address emerging strains. For the WOAH’s Global PPR Eradication Program, the early and efficient detection of all PPRV variants—coupled with proactive containment measures—serves as a critical prerequisite for achieving its objectives. Hence, we established a novel RT-qPCR method, which demonstrated high sensitivity and specificity, capable of detecting all lineages of PPRV.

To enhance the credibility and global applicability of our assay, we acknowledge the importance of benchmarking our method not only against our previously developed N gene-based RT-qPCR assay but also against internationally recognized diagnostic protocols. In future studies, we plan to evaluate the performance of our P gene-based RT-qPCR assay in comparison with the WOAH-recommended assays and other peer-reviewed methods targeting the F or N genes. Including these comparisons would provide a more comprehensive validation of our method’s diagnostic value and allow for broader adoption in different regions and surveillance systems. We also aim to collaborate with international laboratories to test the assay across various genetic backgrounds of PPRV field isolates, thereby further validating its robustness and utility in global eradication efforts.

## Data Availability

The original contributions presented in the study are included in the article/supplementary material; further inquiries can be directed to the corresponding authors.
